# Avian Influenza Virus (H5N1) Was Not Detected Among Dairy Cattle and Farm Workers in Pakistan

**DOI:** 10.1111/irv.13317

**Published:** 2024-05-17

**Authors:** Aftab Ahmed, Samia Saqlain, Arslan Rasool, Shaban Muhammad, Sajid Umar

**Affiliations:** ^1^ Friesland Campina Engro Pakistan Ltd Karachi Pakistan; ^2^ Eastern Scientific Corporation (Private) Limited Lahore Pakistan; ^3^ Huazhong Agricultural University (HZAU) Hubei Wuhan China; ^4^ Division of Natural & Applied Sciences (DNAS) Duke Kunshan University Suzhou China; ^5^ Global Health Research Center (GHRC) Duke Kunshan University Suzhou China

**Keywords:** Avian influenza, H5N1, cattle, Pakistan

Dear Editor,

Influenza A virus (IAV) is a zoonotic pathogen that poses a significant challenge to avian and public health [1]. Migratory waterfowl, such as ducks and geese, are the main reservoir of IAVs and spread IAVs to other domestic birds and animals. Cross‐species transmission of IAV is generally rare, but it can occur, particularly if there are mutations in the virus's genetic code that allow it to attach to receptors in a new host or if a reassortment event occurs when two different influenza viruses infect the same host simultaneously and exchange genetic material. The Avian Influenza A(H5N1) virus, commonly known as “bird flu,” remains primarily a virus that affects birds, but it has on occasion infected humans. A relatively low number of sporadic human infections with A(H5N1) has been reported over the years. A total of 26 sporadic human cases from January 2022 through April 2024 [2] from eight countries indicates ongoing transmission from birds to humans. These cases highlight the potential for this virus to cause severe illness and death in humans. On rare occasions, A(H5N1) has crossed over to some nonavian species especially when there has been close contact with infected birds [3, 4, 5]. Cattle, like other mammalian species, are generally susceptible to specific types of IAV and Influenza D virus. Typically, A(H5N1) is not a common infection in cattle. Surprisingly, numerous sporadic cases of A(H5N1) have been reported recently from dairy cattle in multiple states of the United States [5, 6]. The emerging bovine H5N1 virus is novel to the cattle industry. Unlike other mammals, H5N1 grows in the udder of the dairy cows and does not seem to cause respiratory disease in cattle. Bovine H5N1 virus has undergone a specific adaptation in an enzyme called polymerase allowing better replication inside cow udder. A dairy farm worker with conjunctivitis was also confirmed positive highlighting fresh concerns of bovine H5N1 virus to human health [5, 6]. The dairy worker might have encountered this virus during the milking process or through hand‐to‐eye contact. Further genetic changes within polymerase enzyme or other genome segments of bovine H5N1 virus could allow for faster adaptation and may even support cattle‐to‐cattle or cattle to human transmission [5, 6]. Together, these reports highlight that H5N1 has potential to evolve and become a serious threat to human health. There is no data bovine H5N1 virus from Pakistan. Therefore, this study was designed to monitor the prevalence of IAVs and potential spillover of novel H5N1 among cattle and farm workers in Punjab province of Pakistan. Human subject research was approved by Institutional Review Board (IRB) at Duke Kunshan University, China (2024SU040).

As part of the Influenza D virus surveillance study, we collected nasal washes (*n =* 117) and nasal swab samples (*n =* 376) from farm workers and dairy cattle respectively from January 2023 to March 2024 at six commercial dairy cattle farms located in different cities (Lahore, Kasur, Sahiwal, Multan, Layyah, and Bahawalpur) within Punjab province (Figure 1). These farms were visited twice a month to collect nasal wash and nasal swab samples as described previously [7]. In addition, we also collected milk samples (*n =* 243) during milking process in sterile tubes from different farms in April 2024 to detect bovine H5N1 virus. A commercial RNA extraction Kit (Cat#9766, Takara, Dalian, China) was used to extract viral RNA from samples following the manufacturer's recommendations [1]. To validate the integrity of reagents and the extraction process, we included positive and negative controls. Extracted samples were screened for H5N1 via one step RT‐PCR using HiScript II One Step RT‐PCR Kit (Vazyme, Nanjing, China). Specific primers were used to target hemagglutination (HA) gene [8]. Positive and negative controls were added in each PCR run to confirm the results. All positive controls showed successful amplification while negative controls did not yield amplification during PCR reactions.

**FIGURE 1:**
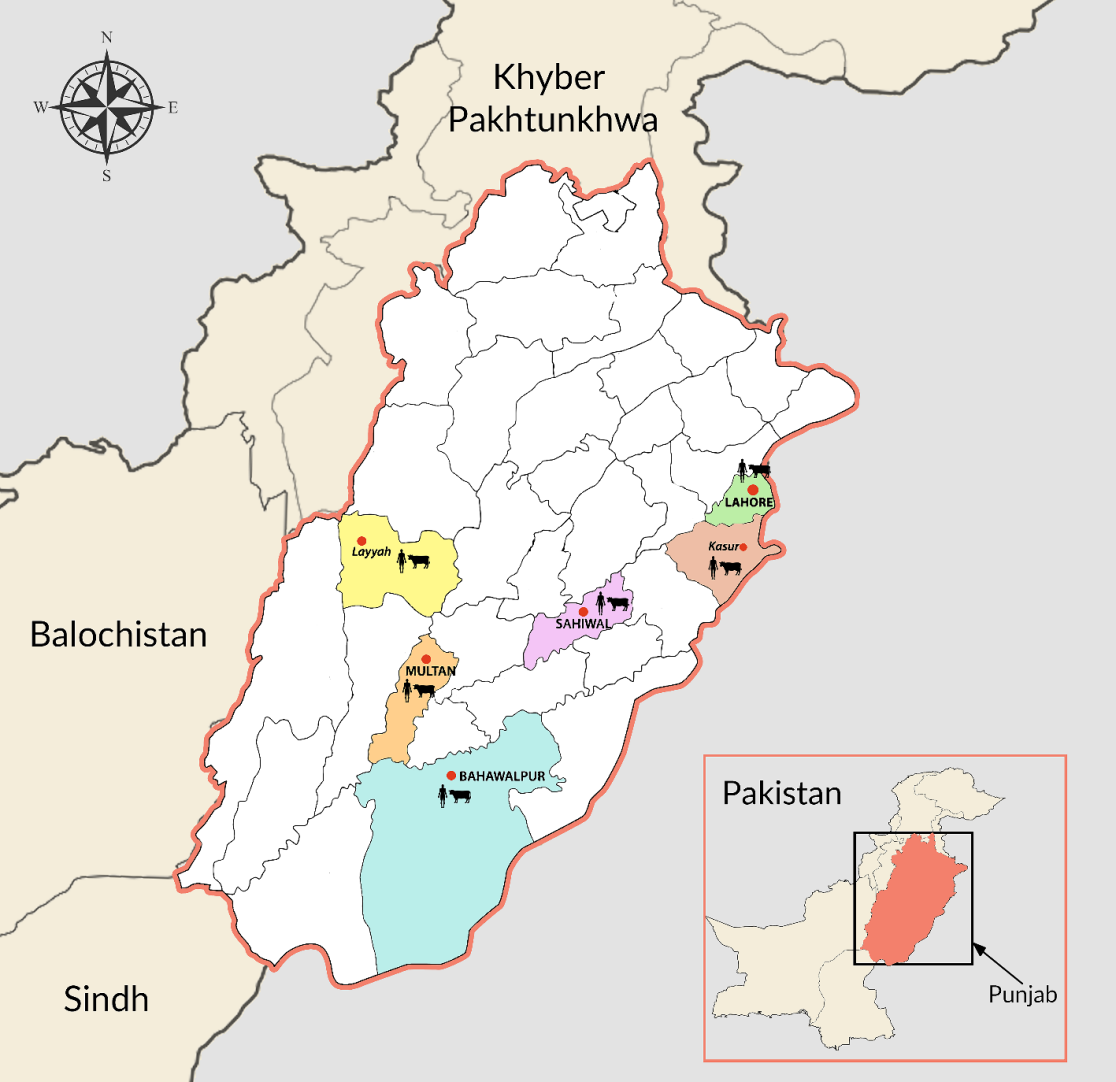
Map of Punjab province in Pakistan, highlighting the cattle farms enrolled in this study. *Source:* www.biorender.com 2024.

No evidence for H5N1 infection was found in any sample type of cattle and farm workers in this study. Assuming our samples were representative of cattle and farm workers in Punjab province during the study time period, H5N1 did not appear to be endemic in Punjab cattle population. These findings could also be linked to strict biosecurity measures at these farms. The inclusion of more cattle farms in sampling plan in other provinces of Pakistan might reverse our observations about H5N1 prevalence. Despite the limitations of small sample size, small geographical area and duration of the study, our study is still valuable as it provided preliminary data with novel methodology that can prompt larger and more comprehensive studies.

To date, bovine H5N1 virus which is currently circulating in US dairy cattle poses a low risk to humans. However, animal farm workers and veterinarians need to be vigilant as they are at higher risk of infection. Appropriate biosecurity measures should be practiced while performing routine work at dairy farms to prevent interspecies transmission of bovine H5N1 virus. Additional sporadic human infections are anticipated because H5N1 has wide circulation in wild and domestic poultry and has potential to evolve rapidly if they are left unchecked. US has been a main exporter of dairy cattle to Pakistan. Therefore, dairy cattle herds must be tested for bovine H5N1 virus before shipment to Pakistan as a preventive measure. It is expected that mammalian host range and zoonotic transmission of IAV will expand due to globalization, intensive trade, and climate change. Therefore, it is critical to exercise proper biosecurity practices at animal farms. Keeping in mind the zoonotic potential and current wave of bovine H5N1 virus among US dairy cattle herds, a One Health approach is urgently required for regular monitoring of IAV in wild birds, domestic poultry, mammals, and people worldwide to determine genetic changes and public health risk.

## Author Contributions

**Aftab Ahmed:** conceptualization, methodology, investigation. **Samia Saqlain:** investigation, formal analysis. **Arslan Rasool:** conceptualization, methodology, investigation. **Shaban Muhammad:** investigation, validation, visualization. **Sajid Umar:** conceptualization, methodology, investigation, validation, formal analysis, supervision, funding acquisition, project administration, writing–original draft, writing–review and editing.

## Conflicts of Interest

The authors declare no conflicts of interest.

### Peer Review

The peer review history for this article is available at https://www.webofscience.com/api/gateway/wos/peer‐review/10.1111/irv.13317.
